# Utility and quality-adjusted life-years in coronary artery disease: Five-year follow-up of the MASS II trial

**DOI:** 10.1097/MD.0000000000009113

**Published:** 2017-12-15

**Authors:** Sara Michelly Gonçalves Brandão, Whady Hueb, Yang Ting Ju, Antonio Carlos Pedroso de Lima, Carisi Anne Polanczyk, Luciane Nascimento Cruz, Rosa Maria Rahmi Garcia, Myrthes Emy Takiuti, Edimar Alcides Bocchi

**Affiliations:** a Instituto do Coracao (InCor), Hospital das Clinicas HCFMUSP, Faculdade de Medicina, Universidade de Sao Paulo, SP, BR; b Institute of Mathematics and Statistics, Department of Statistics, University of São Paulo, São Paulo, SP; c Post Graduate Program in Cardiology and Cardiovascular Disease, Federal University of Rio Grande do Sul; d Instituto Nacional para Avaliação de Tecnologia em Saúde, IATS/CNPq, Porto Alegre, RS, Brazil.

**Keywords:** CABG, coronary artery disease, cost-effectiveness, PCI, quality of life

## Abstract

**Objectives:**

This study evaluated the utility and quality-of-life year measurements for patients with coronary artery disease who underwent any of 3 therapeutic strategies with a 5-year follow-up.

**Methods:**

Quality-of-life data were obtained from the Medicine, Angioplasty, or Surgery Study II trial. To obtain utilities, the 36-Item Short-Form questionnaire was converted to a 6-Dimensional Health State Classification System.

**Results:**

Of the 611 initial patients, 579 completed the questionnaire. In all, 188 patients received the surgical treatment—194 the percutaneous, and the remaining 197 the medical. The median utility scores for the 5 years analyzed were 0.809 (95% confidence interval [CI] 0.794–0.842) for patients assigned to percutaneous coronary intervention, 0.755 (95% CI 0.723–0.774) for medical treatment, and 0.780 (95% CI 0.761–0.809) for coronary artery bypass graft surgery. The difference between percutaneous coronary intervention and medical treatment was statistically significant (*P* < .05, Dunn test). The median cumulative quality-of-life years across the 5 years were 3.802 (95% CI 3.668–3.936) for percutaneous, 3.540 (95% CI 3.399–3.681) for medical, and 3.764 (95% CI 3.638–3.890) for surgery. Additionally, the median quality-of-life years between percutaneous and medical treatment was 0.262 (95% CI 0.068–0.456), between surgery and medical treatment it was 0.224 (95% CI 0.036–0.413), and between surgery and percutaneous coronary intervention it was −0.038 (95% CI −0.221 to −0.146).

**Conclusion:**

Coronary artery bypass surgery and percutaneous coronary intervention were similar regarding cumulative quality-of-life years; however, they were both superior to that of medical treatment. The results presented are valuable data for further cost-utility studies.

## Abstract 2

Supplemental Digital Content is available in the text

## 1 Introduction

Coronary heart disease is estimated to affect 15.5 million people in the United States at a cost of $10.4 billion per year. In 2011, coronary artery disease (CAD) was the most frequent cause of death among Americans, causing more than 375,000 deaths.^[1]^ The optimal therapeutic strategy for multivessel CAD with stable angina and preserved ventricular function has been widely debated. Therapy may consist of medical treatment (MT), percutaneous coronary intervention (PCI), or coronary artery bypass graft (CABG). In general, these interventions are focused on relief of angina symptoms and better exercise tolerance. In addition, they aim at reducing mortality, morbidity, and budgetary impacts. Nonetheless, improvements in health-related quality of life, expressed as utility measures and quality-adjusted life-years (QALYs), are also important targets of treatment.

Utilities are a type of preference-based measure that reflect the relative desirability for a given health status.^[2]^ Utility scores are used as preference weights to calculate QALYs, which are advantageous, because QALY incorporates both the impact of a treatment on a patient's length of life and the impact on their health-related quality of life into a single measure.^[3,4]^ Also, QALYs enable comparisons across different therapies. Because QALYs can be applied to compare different therapies, they have been used frequently and recommended as a summary measure of health outcomes.^[5]^

Contemporary studies have aimed at estimating and comparing utilities and QALYs preferentially in the surgical and percutaneous strategies in multivessel CAD patients.^[6–8]^ The aim of the present study was to report on the utility and QALY measures of PCI, CABG, and MT as the first procedure for the treatment of chronic multivessel coronary disease in the long-term follow-up of a prospective randomized trial—The Medicine, Angioplasty, or Surgery Study (MASS) II.^[9]^

## 2 Methods

**Figure 1:**
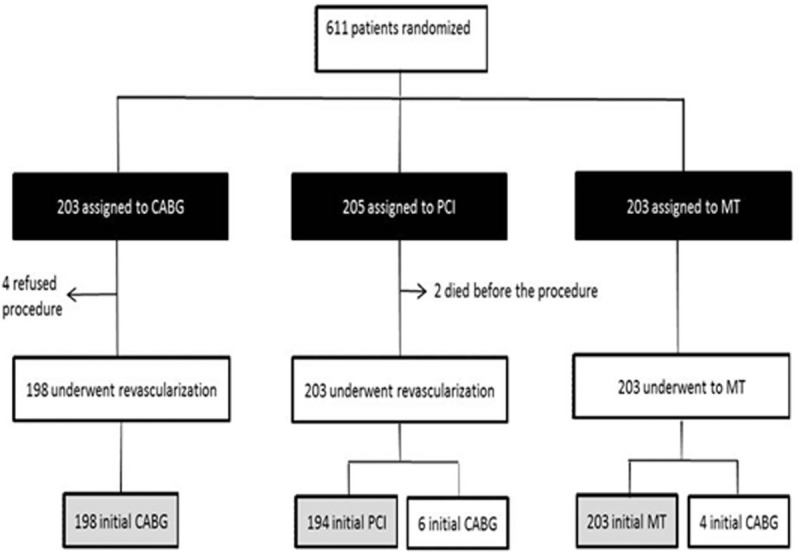
Consolidated Standards of Reporting Trials (CONSORT) diagram. Black boxes represent the intention-to-treat population that was the primary analytical population for this study. The gray boxes represent the per-protocol population. CABG = coronary artery bypass graft, MT = medical treatment, PCI = percutaneous coronary intervention.

Details of the MASS II design, study protocol, patient selection, and inclusion criteria have been reported previously^[9]^ (Fig. 1 and Supplementary File 1).

Briefly, patients with angiographically documented proximal multivessel coronary stenosis >70% by visual assessment and documented ischemia were considered for inclusion. Patients gave written, informed consent and were randomly assigned to a treatment group. The Ethics Committee of the Hospital das Clínicas da Faculdade de Medicina da Universidade da São Paulo approved the trial under no. 264/94/11. From May 1995 to May 2000, 611 patients were randomly assigned to undergo CABG (n = 203), PCI (n = 205), or MT (n = 203).

The 36-Item Short-Form Health Survey (SF-36) version 1 (SF-36V1) was used to assess quality of life at baseline and at 6, 12, 24, 36, 48, and 60 months of follow-up.^[10]^

### 2.1 Preference-based measures

To obtain utilities, the items of the SF-36 were converted into a 6-Dimensional Health State Classification System, the SF-6D. The SF-6D is a single-index summary preference-based measure of health derived from 11 items of the SF-36,^[11]^ allowing a total of 18,000 distinct health states.^[12]^ The domains and SF-36 items used to construct the SF-6D included physical functioning (items 3a, 3b, and 3j), role limitation due to physical problems (item 4c), and emotional problems (item 5b), social functioning (item 10), bodily pain items (items 7 and 8), mental health (items 9b and 9f), and vitality (item 9e). The SF-6D algorithm generates health status values using a representative and validated sample of the Brazilian general population from the capital city of Rio Grande do Sul^[13]^ to approximate the societal viewpoint. This general population approach is consistent with guidance provided by health technology assessment agencies in Brazil.^[14]^ The health state utility score ranges from 0 to 1, where 0 represents death and 1 represents perfect health.^[3,15]^

### 2.2 QALYs

For each patient, the area under curve (AUC) approach was obtained manually by calculating the average utility values between 2 consecutive time measurements and multiplying it by the time interval between the measurements, and summing up all the values.^[16,17]^

The QALY cumulative was measured by summing the QALY of all periods across 5 years of follow-up.

The QALYs obtained were calculated as the difference in the mean QALYs for 1 strategy compared with the next less-effective alternative strategy.

### 2.3 Ethics committee approval

All patients provided written informed consent and were assigned to a treatment group. The Ethics Committee of the Heart Institute of the University of São Paulo Medical School, São Paulo, SP, Brazil, approved the trial. All procedures were performed in accordance with the Declaration of Helsinki.

### 2.4 Statistical analysis

The SF-6D algorithm requires the respondent to complete most of the questions from the SF-36 to calculate a health-state utility. Omitting crucial responses for the calculation of utility means the remaining responses cannot be used for this purpose; thus, missing data for utility and QALYs due to missing items (one or more missing answers to questions within a questionnaire) and missing forms (the whole questionnaire is missing for a patient) at a set time interval were imputed. Because the National Institute for Health and Clinical Excellence does not specifically mention how missing data should be approached,^[18,19]^ we performed a multiple imputation adjusted for age, sex, previous myocardial infarction (MI), and diabetes mellitus to replace the individual missing value for utility.

Surviving individuals with only baseline values or no information on quality of life were not included. Fatal cases were censored at the date of death. We performed this crude analysis (undiscounted), because it was based on long-term primary data with each future-time interval not being obtained by a projection.

Measurement data based on intent to treat are reported as frequencies with percentages for all categorical variables, as mean ± standard deviation (SD) for normally distributed continuous variables, and as mean ± SD for median with 95% confidence intervals (CIs) of the medians based on 5000 replications for utility. Kolmogorov-Smirnov tests were used to test the normal distribution of the data. Furthermore, we controlled for imbalance in baseline utility values in the estimation of mean QALYs in each group and mean differential QALYs by regression analysis.^[20]^ QALYs and differential QALYs over 5 years are reported as means, with 95% CIs of the mean based on 5000 replications.

For categorical variables, we used the chi-square test to compare the 3 groups. All comparisons of utility values were conducted with nonparametric tests due to the non-normal distribution of these values. The Kruskal-Wallis tests obtained via bootstrapping with 95% CIs based on 5000 replications were used to assess differences between variables. Significant results demonstrated by the Kruskal-Wallis tests were further analyzed for significance with Dunn test. Survival data were estimated with the Kaplan-Meier method, and differences among groups were assessed using the log-rank test.

Friedman and Wilcoxon 2-sample signed-rank tests were used to compare the relative changes of utilities among the different time points. A Bonferroni-corrected alpha level was applied by dividing the alpha value by the number of comparisons. Statistical differences for pair-wise comparisons with a value of *P* < .007 were considered significant. Differences were considered statistically significant when the 95% CIs did not overlap 1.0 or when *P* < .05 (2-sided test). All statistical analyses were performed with SPSS 21.0 software and the R program.

The smallest difference in score that the patient perceived as beneficial was called the minimal important difference (MID), and its concept was developed to better express clinically important benefit or deterioration rather than just statistically significant differences or changes in patient-reported outcomes.^[21,22]^ As an additional analysis, differences in the utility score over time and between groups were considered clinically relevant for median differences of 0.037 based on a previous study.^[12]^

## 3 Results

### 3.1 Baseline clinical characteristics

In all, 611 patients with multivessel CAD were randomized to receive PCI (205), CABG (203), or MT (203) as the first approach at the time of randomization. Surviving individuals with only baseline values and incomplete information on quality of life were not included (n = 32; PCI = 11, GABG = 15, MT = 6), leaving 579 patients in this study. Patients were mostly similar across groups, but prior MI was more frequent in the PCI group (*P* *=* .047). In this sample, the MT group had a higher incidence of a history of diabetes (*P* = .012). Table 1 summarizes the baseline characteristics of the patients.

**Table 1:**
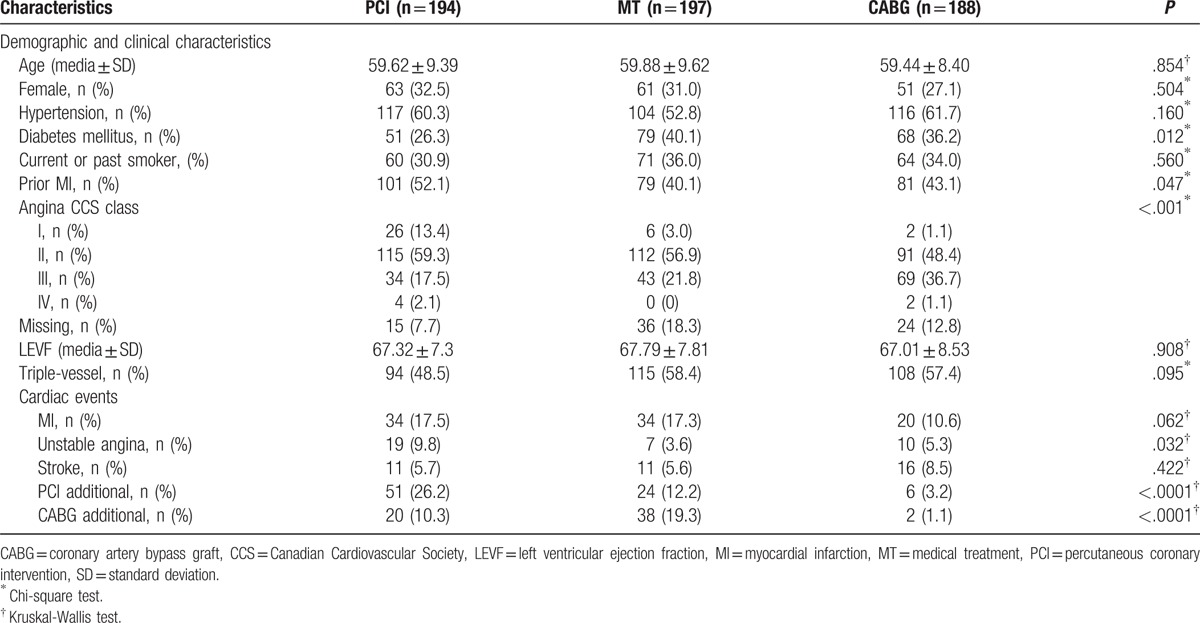
Baseline characteristics and cardiac events at the 5-year follow-up.

### 3.2 Follow-up outcomes

**Figure 2:**
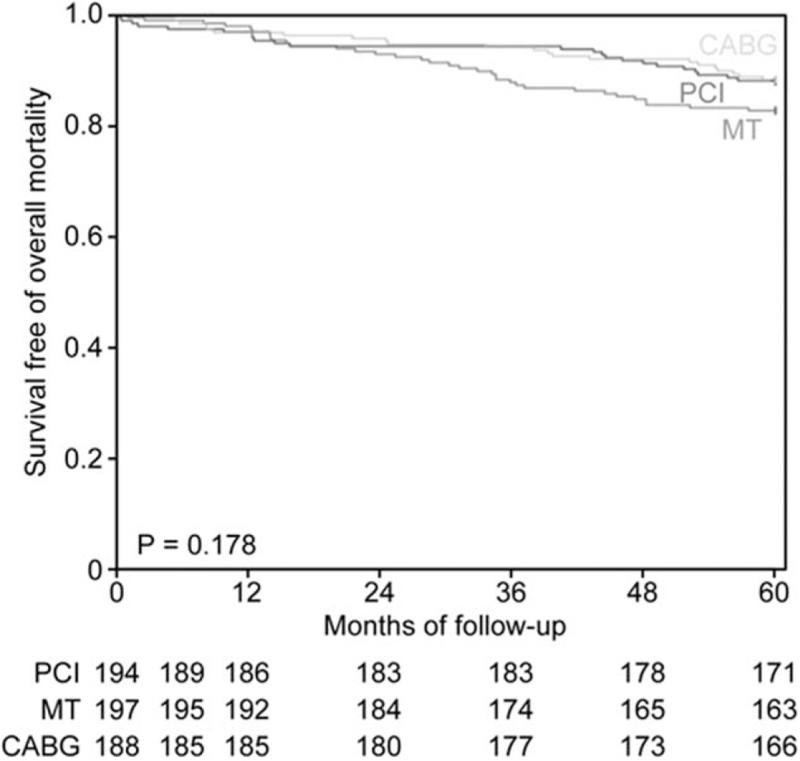
Probability of survival free of overall mortality among patients in the medical treatment (MT), percutaneous coronary intervention (PCI), and coronary artery bypass graft (CABG) groups.

All patients received medical regimens according to a predefined approach. No surviving patient was lost to follow-up. The minimal duration of follow-up was 5 years. No differences existed among the cumulative overall mortality curves associated with the 3 therapeutic strategies (*P* = .178; Fig. 2).

Adverse cardiac events at the 5-year follow-up are shown in Table 1. The patients allocated to the MT group had a lower incidence of unstable angina (3.6%; *P* = .032). There was a significant difference among the groups in the frequency of additional PCI (*P* < .001) and additional CABG (*P* < .001).

### 3.3 Utility

**Figure 3:**
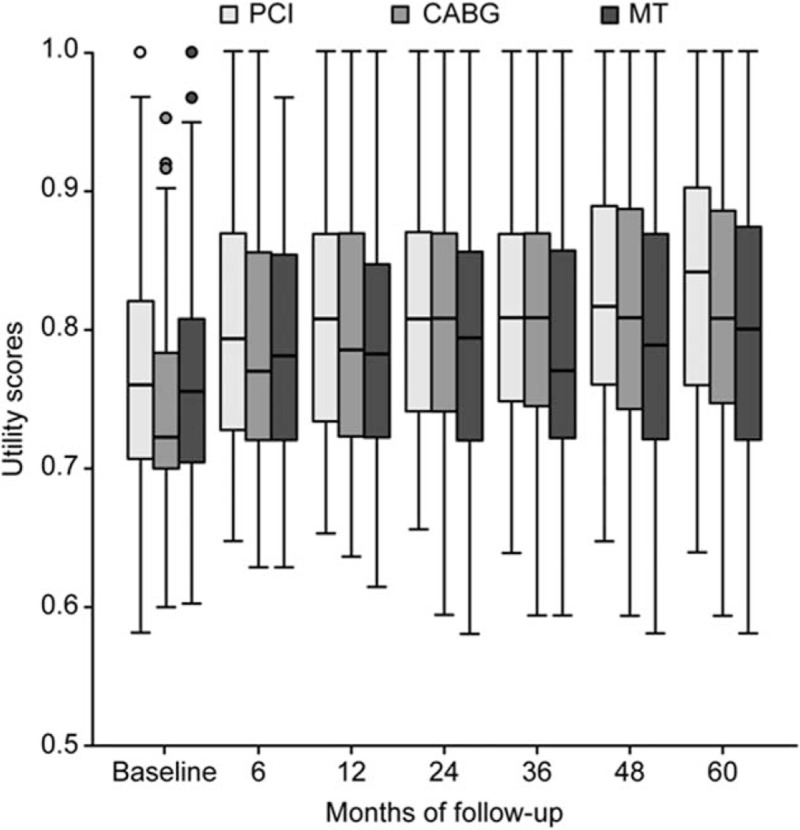
Box-plot graph showing medians and interquartile ranges of utility evaluation of the medical treatment (MT), percutaneous coronary intervention (PCI), and coronary artery bypass graft (CABG) patients over the course of the trial.

At baseline, the median utility score in the PCI group was significantly higher than that in the CABG group (0.76 vs 0.72, respectively; *P* < .05, Dunn test); the difference in the utility score was 0.038. No significant differences occurred between the MT group and the PCI or CABG groups (Fig. 3). Utility values are summarized in Table 2, and utility-difference scores are provided in the Supplementary File, Table 1 (http://links.lww.com/MD/C17).

**Table 2:**
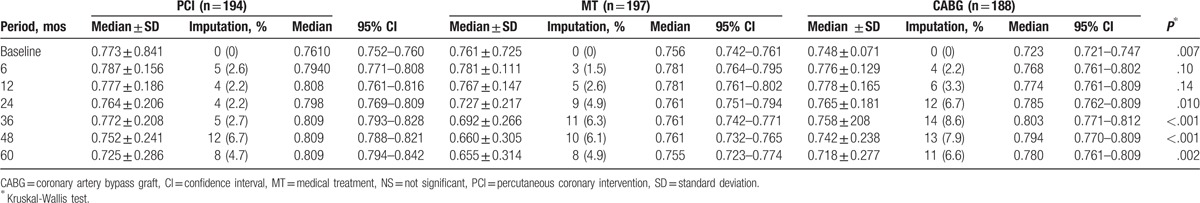
Utility of treatments.

The changes in measures through all follow-up periods shown for all groups were only significant between baseline and the 6-month follow-up (*P* < .001, Bonferroni-corrected), and the greatest changes in scores were observed in the CABG group (0.045).

No statistical differences were found among the groups at 6 and 12 months, and although median utility at the 6-month follow-up was higher after PCI or CABG (0.79 vs 0.77, respectively), the change in utility score was only 0.026. For the second year, the median utility remained higher than the previous utility for the CABG group; however, differences were observed only between the MT and PCI groups. MT utilities were significantly lower than in the PCI group (0.78 vs 0.80, respectively; *P* < .05, Dunn test), and the utility difference score was 0.037.

During the subsequent years of follow-up, patients assigned to the PCI group continued to experience no significant improvement. Nevertheless, the MT and CABG groups experienced subtly lower scores than in the second year, and significant differences were observed among MT and the other 2 groups (*P* < 0.05, Dunn test) at all subsequent time intervals, except at 60 months, when the difference was just between the PCI and MT groups (0.809 vs 0.755, respectively; *P* < .05, Dunn test). Overall, median utility improved significantly for PCI and CABG groups over the course of the trial (*P* *=* .003 and *P* < .001, respectively, Bonferroni-corrected). The difference scores at 36, 48, and 60 months between PCI and MT were 0.048, 0.048, and 0.054, respectively. It should be noted that the change in utility score between MT and CABG was 0.042 at 36 months.

### 3.4 QALYs

The mean cumulative QALY measurements across the 5 study-years were 3.802 (95% CI 3.668–3.936) for the PCI group, 3.540 (95% CI 3.399–3.681) for the MT group, and 3.764 (95% CI 3.638–3.890) for the CABG patients.

Additionally, the mean QALYs gained between the PCI and MT groups was 0.262 (95% CI 0.068–0.456), between the CABG and MT groups 0.224 (95% CI 0.036–0.413), and between the CABG and PCI groups −0.038 (95% CI −0.221 to −0.146).

## 4 Discussion

To the best of our knowledge, this is the first study to estimate and compare utility and QALY measurements among symptomatic patients with multivessel CAD who underwent CABG, PCI, or MT in a prospective randomized trial. Also, this study is particularly unique, because of the long-term follow-up period that could assess long-term clinical outcomes. Our results showed that PCI and CABG as initial treatments were associated with higher utility and QALYs compared with MT. However, during the follow-up, utility increased in all groups.

Our overall results are not directly comparable with results of previous studies, because we applied this research tool in a study that compared the 3 therapeutic strategies simultaneously. In addition, long-term follow-up is not routine in clinical practice. On the contrary, our results are consistent with previous studies that compared PCI with bare-metal stents and CABG.^[6–8]^ The results for a 1-year follow-up randomized study with almost 70% of patients having single-vessel stable CAD were comparable between the PCI and off-pump CABG groups on the EuroQol 5-dimension (EQ-5D) questionnaire at 12 months. Although the therapeutic strategies were similar, the quality-of-life instrument used by these authors does not include all the dimensions offered by our study. In this study, QALYs in the PCI group were comparable with CABG at 1-year follow-up, similar to our QALYs for the PCI and CABG groups across 5 years of follow-up,^[6]^ but in a smaller sample than that of MASS II and with a shorter follow-up time. The Stent or Surgery trial (SoS) reported no difference in the EuroQol 5-dimension questionnaire (EQ5D) utility at 6 months and 1 year of follow-up, and found QALY values that were comparable between the PCI and CABG groups after 1 year, similar to our study across 5 years of follow-up.^[7]^ Our findings differ from the Study of Economics and Quality of Life (SEQOL) results in which CABG with or without extracorporeal circulation and PCI were compared for 10 years.^[8]^ In this previous study, utility was more favorable among CABG patients for the first year, which was different from our results, although the values became similar thereafter.^[8]^ In the drug-eluting stent (DES) era, comparing previous studies of DES-PCI versus CABG for patients with multivessel CAD, the Future Revascularization Evaluation in Patients with Diabetes Mellitus: Optimal Management of Multivessel Disease (FREEDOM) trial with predominant paclitaxel-eluting stent (PES), and the Synergy Between Percutaneous Coronary Intervention with TAXUS and Cardiac Surgery (SYNTAX) trial with DES, both first-generation DES, reported no difference in EQ5D utility from 6 months through 5 years of follow-up. In our study, differences were also not observed across 5 years of follow-up.^[23,24]^ The QALY values were slightly higher for CABG at 5 years,^[23,24]^ which was in contrast with our results in which PCI was discreetly but not significantly higher than CABG. These findings suggest that DES-PCI may not provide additional benefits compared with conventional PCI.

Concerning the comparison between bare-metal stent PCI and MT, the Clinical Outcomes Utilizing Revascularization and Aggressive druG Evaluations (COURAGE) trial reported results discreetly consistent with our findings. However, the clinical characteristics of the COURAGE population were slightly different, because of the inclusion of 34% single-vessel stable CAD patients who had the best prognosis and a standard gamble method used to measure utility.^[25]^ PCI utility results in the COURAGE trial were significantly higher in comparison with the MT utility results at the third year of follow-up, whereas our results showed differences from the second year that persisted thereafter.^[25]^ To our knowledge, no early studies compare CABG versus MT.

The reason for the similarity of utility with PCI compared with CABG and the superiority of utility to MT in an intent-to-treat analysis during the follow-up period in our results highlights a challenging issue related to the higher incidence of unstable angina. Consequently, this unstable angina required additional PCI in the PCI group in comparison with MT and CABG. A higher incidence occurred of AMI and additional CABG in comparison with that in the CABG group. However, despite the greater possibility of complete revascularization and the higher effectiveness in relieving angina, CABG was associated with chronic issues related to surgery, such as persistent thoracic pain after thoracotomy secondary to surgical trauma sequelae. Also, the manifestation or progression of coronary disease or bypass occlusion after CABG possibly hampers the decision-making process regarding subsequent interventions, because physicians may have a higher threshold for recommending repeat revascularization after CABG. Factors such as coronary anatomy after CABG with the frequent necessity of intervention through bypass due to the higher incidence of occlusion of native arteries with previous stenosis, the historical credibility of surgery, and the discomfort and risk of a second surgical trauma may contribute to the decision process. On the contrary, PCI patients could have more opportunities for repeated intervention or for the evolution of the disease in other coronary artery regions needing intervention. In addition, PCI is a less invasive revascularization procedure, requires a shorter recovery period, and causes less acute and chronic postoperative complications and comorbidities. Patients who receive MT may also have opportunities for new additional procedures, such as PCI or CABG, although CABG was the most indicated intervention. Additionally, the improvement in pharmacological treatment of CAD might contribute to better explaining the results observed in MT patients.

Our study has some limitations. First, interpretation of the results may be affected by subsequent innovations, although previous studies do not support this.^[6–8,25]^ Second, 0.05% of patients were excluded either for incomplete questionnaires, no information on quality of life, or no post baseline assessment, although the conclusion of this study was unlikely to have been affected by these missing data. Third, SF-6D health state preference values were measured in a single-center study of the Brazilian population and may have been influenced by Brazilian sociocultural characteristics, although our results were consistent with results of international studies. In addition, we did not provide a utility measure at the time of the events or subsequent procedures.

On the contrary, unicentric studies allow the questionnaires to be drawn up homogeneously. Also, a 5-year follow-up period might be considered insufficient to evaluate CAD events. However, longer evolution studies may include different morbidities and include factors that confound prognoses. Additionally, studies with 10 years of follow-up have also shown no difference in mortality—1 of the components used to calculate QALYs.^[26]^ As another limitation, interpretation according to the MID showed that utility measures for each group increased across the 5 study-years, although the between-group utility score at 60 months was only considered important when comparing PCI and MT. However, it is important to point out that MID has not been established to discriminate between groups.^[12,22,27]^ Furthermore, as a long-term follow-up study, procedures were performed using standard techniques from the beginning of the study; however, recent 2014 American College of Cardiology/American Heart Association and 2013 European Society of Cardiology guidelines for CAD management support new treatment and pharmacological options similar to those used in our study.^[28,29]^ Finally, our study was a retrospective review of medical records, although we obtained consistent data.

Our study will have implications for the implementation of future cost-utility analyses of multivessel CAD therapies, and our results provide information to regulatory agencies for decision-making processes. Additionally, to the best of our knowledge, this is the first study to demonstrate preference-based utility and QALY measurements among patients with multivessel CAD undergoing 1 of 3 common treatment strategies for this disease over the long term.

## 5 Conclusions

Considering the health-related quality-of-life measurement as lending support to the decision-making processes, PCI and CABG were shown to be the treatments with higher cumulative QALYs among multivessel CAD patients compared with MT, but no difference existed between PCI and CABG.

## Supplementary Material

Supplemental Digital Content
