# Supplementary material for: Utility and quality-adjusted life-years in coronary artery disease: Five-year follow-up of the MASS II trial
Source: Medicine (Baltimore). 2017 Dec 15;96(50):e9113. doi: 10.1097/MD.0000000000009113 (PMC5815720; doi:10.1097/MD.0000000000009113)
Supplement: Supplemental Digital Content [file medi-96-e9113-s001.docx]

# Differences in median utility scores within groups and between groups

| **MONTHS** | **GROUPS** | | |
| --- | --- | --- | --- |
| **Time interval ^*^** | **PCI** | **MT** | **CABG** |
| 0 - 6 | 0.033 | 0.025 | 0.045 |
| 6 - 12 | 0.014 | 0.000 | 0.006 |
| 12 - 24 | 0.010 | 0.020 | 0.011 |
| 24 - 36 | 0.011 | 0.000 | 0.018 |
| 36 - 48 | 0.000 | 0.000 | 0.009 |
| 48 - 60 | 0.000 | 0.006 | 0.014 |
| 0 - 60 | 0.081 | 0.755 | 0.780 |
| **Period**^†^ | **MT vs. CABG** | **MT vs. PCI** | **PCI vs. CABG** |
| **0** | -0.033 | 0.005 | 0.038 |
| **6** | -0.013 | 0.013 | 0.026 |
| **12** | 0.007 | 0.027 | 0.034 |
| **24** | 0.024 | 0.037 | 0.013 |
| **36** | 0.042 | 0.048 | 0.006 |
| **48** | 0.033 | 0.048 | 0.015 |
| **60** | 0.025 | 0.054 | 0.029 |

* Given by the difference of the median utility scores by group in a subsequent time interval; † Given by the difference of the median utility scores between 2 groups for each period.
